# Comparative study of generalized couette flow of couple stress fluid using optimal homotopy asymptotic method and new iterative method

**DOI:** 10.1038/s41598-021-82746-8

**Published:** 2021-02-10

**Authors:** Muhammad Farooq, Alamgeer Khan, Rashid Nawaz, Saeed Islam, Muhammad Ayaz, Yu-Ming Chu

**Affiliations:** 1 Department of Mathematics, Abdul Wali Khan University, Garden Campus, Mardan, KP Pakistan; 2 Department of Mathematics, Huzhou University, Huzhou, 313000 China; 3 Hunan Provincial Key Laboratory of Mathematical Modeling and Analysis in Engineering, Changasha, University of Science and Technology, Changasha, 410114 China

**Keywords:** Applied mathematics, Computational science

## Abstract

In this research work, we have studied the steady generalized Couette flow of couple stress fluid between two parallel plates considering the non-isothermal effects. The governing equations that are, continuity, momentum and energy equations are reduced to ordinary differential equations. The Optimal Homotopy Asymptotic Method (OHAM) and New Iterative Method (NIM) are used to solve this coupled system of differential equations. Using the said methods, we have obtained expressions for velocity profile, temperature distribution, volume flux, average velocity and shear stress. The results of OHAM and NIM are compared numerically as well as graphically and a tremendous agreement is attained.

## Introduction

In the last few decades, non-Newtonian fluids attained more importance for scientists and researchers due to the wide range of applications in numerous industrial and engineering processes^1–8^. Stokes^9^ has proposed the theory of couple stress fluids in fluid mechanics, which appealed scientists and researchers. In classical theory, the Couple stress fluid theory is the generalization of the viscous Newtonian fluids. The concept of couple stresses arises because of the methodology, that how the fluid medium is modelled for the mechanical interactions. This theory effectively described the flow behaviour of fluids consisting of a substructure such as liquid crystals, animal blood and lubricants with polymer additives^10,11^.

Among the several models, which described the Non-Newtonian behaviour established by certain fluids, the couple stress fluid attained surprising attention^5,9,12–18^. The couple stress fluid denotes those fluids which consist of rigid and randomly oriented particles suspended in a viscous medium. The stress tensor is not symmetric in those fluids, thus in the classical Newtonian theory, exact flow behaviour cannot be estimated. The remarkable characteristic of the couple stress fluid model is that, it outcomes in the form of differential equations which are similar to the Navier–Stokes equations. The said model has been extensively used because of its relative mathematical simplicity as related to other models established for the fluid under consideration. The study of heat transfer flow is very important in various engineering applications, for example, radial diffusers; drag reduction, the design of thrust bearing, transpiration cooling, and thermal recovery of oil. The heat transfer has a key role in handling and processing of non-Newtonian mixtures^19–21^. Researchers in^22,23^ have explored different problems of couple stress fluids flow past axisymmetric bodies. Due to the nonlinear behaviour of the governing equations of the flow of couple stress fluid and having higher order than Navier–Stokes equations, it is very difficult to find the exact solution.

In literature, different techniques have been applied to explore flow problems. The key tools to find the solutions of flow problems are numerical methods, perturbation methods, iterative methods and homotopy based methods. Each approach has its own pro and cons. Discretization is used in numerical techniques which affect the accurateness. These methods required a lot of computational work and time. When strong non-linearity occurs in problems the numerical methods do not provide us more accurate results. Perturbation methods have some limitations as well, such as small parameter assumption and strong nonlinearity. Iterative methods are sensitive to initial condition and the accuracy of these methods improves as the number of iterations increases. The well-known iterative methods for solutions of differential equations are New perturbation iteration method^24^, optimal iteration method^25^ and an iteration procedure with application to Van der Pol oscillator, which is a powerful tool used to determine the periodic solutions of non-linear equations^26^ etc.

Optimal homotopy asymptotic method (OHAM) for the solutions of differential equations is one of power homotopy based techniques. This technique was introduced by Marinca et al.^27–31^ for the solution of differential equations. This method does not require discretization like other numerical methods which is time consuming and is valid in the absence of small or large parameter, unlike other perturbation methods. The method is also free from an initial guess, unlike the iterative methods. Furthermore the convergence of the method is controlled by more flexible function called the auxiliary function. One of the drawback of OHAM is that the values of auxiliary convergence control parameters in OHAM are calculated using method of least squares which become more time consuming in case of high nonlinear problems. An alternate way is to use collocation method, but in the later case one would compromise on the accuracy. This technique has been employed in different problems^32–40^. Researchers in^41–45^ transformed the highly nonlinear partial differential equations (PDE’s) prescribed by heat flux situations into structure ordinary differential equations (ODE’s) with proper constraints and after that explored using (OHAM). Authors in^46^ investigated a third grade non-Newtonian blood in porous arteries using OHAM. Authors in^47^ explored the steady, laminar, incompressible and two dimensional micro-polar flow employing (OHAM), the authors in^48^ reduce the steady, laminar, incompressible, and two-dimensional micro-polar flow to a set of nonlinear boundary value problem using suitable similarity transformations and explored using OHAM. Authors in^49^ investigated heat transfer in the air heating flat plate solar collectors using OHAM and HPM.

In 2006 Daftardar-Gejji and Jafari have proposed a new method known as the new iterative method (NIM) for solutions of linear as well as non-linear equations^50^. The proposed method handles linear and nonlinear equations in an easy and straightforward way.NIM does not require the need for calculation of tedious Adomian polynomials in nonlinear terms like ADM, the need for determination of a Lagrange multiplier in its algorithm like VIM, and the need for discretization like numerical methods. The method is also free from small parameter assumption unlike reguler perturbation method. As NIM is an iterative method so it requires an initial condition to start which is its main disadvantage. Afterwards, the method was extended by many researchers for different problems including algebraic equations, evolution equations, ordinary and partial differential equations and system of non-linear dynamical equations. Authors in^51^ investigated linear as well as nonlinear fractional diffusion-wave equations on finite domains with Dirichlet boundary conditions using a new iterative method, linear and non-linear fractional diffusion-wave equations have been explored using New Iterative Method^52^. Authors in^53^ explored a fractional version of logistic equation using new iterative method. In^54^ authors have been used a new iterative method to present, sufficiency conditions for convergence of decomposition method. The new iterative method has been employed to solve the nth-order linear and nonlinear integro-differential equations^55^. Authors in^56^ proposed an efficient modification of new iterative method for the solution of linear and nonlinear Klein Gordon equations. The Newell-Whitehead-Segel equation has been investigated using NIM^57^. Authors In^58^ have suggested a iterative methods for solving nonlinear equations involving only first derivative of the function. A family of iterative methods for solving the system of nonlinear equations has been presented^59^. Authors in^60^ have suggested and analyzed some unique recurrence relations which generate different classes of iterative techniques for investigating nonlinear problems using the coupled system of equations. In^61^ the nonlinear Baranayi and Roberts model has been investigated using NIM. As compared to other analytical techniques, NIM provides solutions with rapid convergence.

In this research work, we have explored the steady generalized plane Couette flow of couple stress fluid between two parallel plates under the influence of non-isothermal effects. OHAM and NIM are employed to solve the coupled system of differential equations.

This paper consists of six sections. “Introduction” section  is devoted to a brief introduction. “Basic governing equations and problem formulation” section consists of basic governing equations and problem formulation. “Description of the methods” section consists of basic ideas of the methods and in “Solution of the problem” section solutions of the problem are given. “Results and discussion” section consists of results and discussions and conclusion of the paper is given in the last section.

## Basic governing equations and problem formulation

### Basic governing equations

The basic equations in^62–67^ of momentum, conservation of mass and energy for an incompressible fluid are as under,

1 $$ \nabla .{\mathbf{W}} = 0, $$

2 $$ \rho \mathop {\mathbf{W}}\limits^{ \bullet } = \nabla .\tau - \eta \nabla^{4} {\mathbf{W}} + \rho f, $$

3 $$ \rho c_{p} \mathop \Theta \limits^{ \bullet } = \kappa \nabla^{2} \Theta + \tau L, $$

where ${\mathbf{W}}$ is the velocity vector, the temperature is denoted by $\Theta$ , $f$ is the body force per unit mass, $\rho$ is the constant density, the thermal conductivity is denoted as $\kappa$ , $c_{p} ,$ is the specific heat, the Cauchy stress tensor is represented by $\tau$, the gradient of ${\mathbf{W}}$ is represented by $L,$ the couple stress parameter is denoted by $\eta$ and the material derivative is denoted by $\frac{D}{Dt}$ and defined as under

4 $$ \frac{D}{Dt}( * ) = \left( {\frac{\partial }{\partial t} + {\mathbf{W}}.\nabla } \right)( * ). $$

The Cauchy stress tensor is denoted and defined as

5 $$ \tau = - pI + \mu A_{1} . $$

where dynamic pressure is represented by $p$ , $I$ is the unit tensor, the coefficient of viscosity is denoted by $\mu$ and $A_{1}$ is the first Rivilin-Ericksen tensor, defined as

6 $$ A_{1} = L + L^{t} . $$

where $L^{t}$ is the transpose of $L.$

### Problem formulation

**Figure 1:**
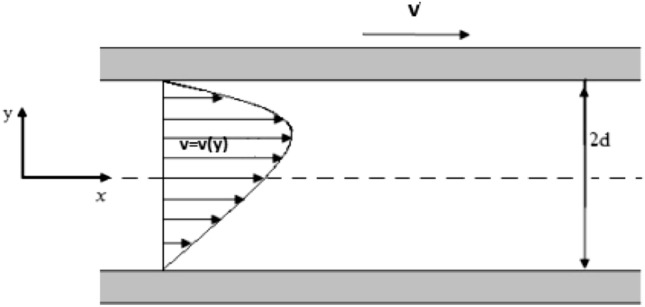
Geometry of generalized plane Couette flow.

Consider a steady flow of an incompressible couple stress fluid between two infinite parallel plates, separated by distance $2d,$ as shown in Fig. 1. Suppose the upper plate is moving with constant velocity ${\mathbf{W,}}$ where the temperature of the lower plate is $\Theta_{0}$ and the upper plate is $\Theta_{1} .$ Both plates are located in the orthogonal coordinates system $\left( {x,y} \right),$ at $y = - d$ and $y = d,$ the motion of the fluid is in the $x - {\text{axis}}$ and the $y - {\text{axis}}$ is perpendicular to the plates. The pressure gradient is zero, $\mu$ is taken to be a function of $\Theta \left( y \right),$ velocity and temperature fields are considered as under

7 $$ {\mathbf{W}} = {\mathbf{W}}\left( {\nu ,\,0,\,0} \right),\,\,\nu = \nu \left( y \right)\,\,{\text{and }}\,\Theta = \Theta \left( y \right). $$

Equation (1) is identically satisfied, if body force is ignored, Eqs. (2)–(3), becomes

8 $$ \mu \frac{{d^{2} \nu }}{{dy^{2} }} + \frac{d\mu }{{dy}}\frac{d\nu }{{dy}} - \eta \frac{{d^{4} \nu }}{{dy^{4} }} + A = 0, $$

9 $$ \frac{{d^{2} \Theta }}{{dy^{2} }} + \frac{\mu }{\kappa }\left( {\frac{d\nu }{{dy}}} \right)^{2} - \frac{\eta }{\kappa }\left( {\frac{{d^{2} \nu }}{{dy^{2} }}} \right)^{2} = 0. $$

where $A = \frac{dp}{{dx}},$ the pressure gradient, is a negative constant.

The boundary conditions for Eqs. (8)–(9), are

10 $$ \nu \left( { - d} \right) = 0,\,\nu \left( d \right) = V, $$

11 $$ \nu ^{\prime\prime}\left( { - d} \right) = 0,\,\,\nu ^{\prime\prime}\left( d \right) = 0, $$

12 $$ \Theta \left( { - d} \right) = \Theta_{0} ,\,\Theta \left( d \right) = \Theta_{1} . $$

Equations (10) represent no-slip boundary conditions. Equations (11) show that couple stresses are zero at the plates. Inclusive, the dimensionless parameters are:

$$ v^{*} = \frac{v}{V},\,y^{*} = \frac{y}{d},\,\Theta^{*} = \frac{{\Theta - \Theta_{0} }}{{\Theta_{1} - \Theta_{0} }},\,\mu^{*} = \frac{\mu }{{\mu_{0} }}, $$

$$ B = d\sqrt {\frac{{\mu_{0} }}{\eta }} ,\,\lambda = \frac{{\mu_{0} \,V^{2} }}{{\kappa \left( {\Theta_{1} - \Theta_{0} } \right)}},\,\,\,A^{*} = \frac{{Ad^{4} }}{V\eta }. $$

Hence Eqs. (8)–(9) with boundary conditions (10)–(12) and ignoring the asterisks can be written as,

13 $$ \frac{{d^{4} \nu }}{{dy^{4} }} - B^{2} \mu \frac{{d^{2} \nu }}{{dy^{2} }} - B^{2} \frac{d\mu }{{dy}}\frac{d\nu }{{dy}} + A = 0, $$

14 $$ \frac{{d^{2} \Theta }}{{dy^{2} }} + \lambda \mu \left( {\frac{d\nu }{{dy}}} \right)^{2} + \frac{\lambda }{{B^{2} }}\left( {\frac{{d^{2} \nu }}{{dy^{2} }}} \right)^{2} = 0, $$

15 $$ \nu \left( { - 1} \right) = 0,\,\nu \left( 1 \right) = 1,\,\nu ^{\prime\prime}\left( { - 1} \right) = 0,\,\nu ^{\prime\prime}\left( 1 \right) = 0, $$

16 $$ \Theta \left( { - 1} \right) = 0,\,\,\Theta \left( 1 \right) = 1. $$

The dimensionless form of the Reynolds viscosity^68–72^ is as follows

17 $$ \mu = \exp \left( { - M\Theta } \right). $$

Let us suppose $M = \varepsilon {\text{m}},$ where $\varepsilon$ is a small parameter. Using Taylor series expansion of Eq. (17) we have

18 $$ \mu = 1 - \varepsilon {\Theta},\,\frac{d\mu }{{dy}} \cong - \varepsilon {\text{m}}\frac{d\Theta }{{dy}}. $$

## Description of the methods

### Basic notion of OHAM

The overview of Optimal Homotopy Asymptotic Method (OHAM) is given in this sub-section let us consider the differential equation

19 $$ L({\mathbf{W}}(x)) + f(x) + N({\mathbf{W}}(x)) = 0,\,\,\,\,\,B\left( {{\mathbf{W}},\frac{{d{\mathbf{W}}}}{dx}} \right) = 0. $$

Here the linear operator is denoted by $L,$ $f(x)$ is known function, ${\mathbf{W}}(x)$ is the unknown function, $N({\mathbf{W}}(x))$ is a non-linear operator and boundary operator is represented by $B.$

Applying OHAM we obtained^30^

20 $$ (1 - r)[L({\mathbf{W}}(x,r)) + f(x))] = H(r)[L({\mathbf{W}}(x,r)) + f(x)) + N({\mathbf{W}}(x,r))],\,\,\,B\left( {{\mathbf{W}}(x,r),\frac{{d{\mathbf{W}}(x,r)}}{dx}} \right) = 0. $$

where the embedding parameter is represented by $$r \in [0,1],[0,1]$$$$,$$ the auxiliary function $$H(r)$$ such that for $$r \ne 0,$$ is non-zero and for $$r = 0$$ i.e. $$H(0) = 0,$$ obviously when $$r = 0$$ and $$r = 1$$ it gives

21 $$ {\mathbf{W}}(x,0) = {\mathbf{W}}_{0} (x),\,\,\,{\mathbf{W}}(x,1) = {\mathbf{W}}(x). $$

The solution ${\mathbf{W}}(x,r)$ varies from ${\mathbf{W}}_{0} (x),$ to ${\mathbf{W}}(x),$ as $r$ varies from $0$ to $1,$ we obtained ${\mathbf{W}}_{0} (x)$ by substituting $r = 0$ in Eq. (19)

22 $$ L({\mathbf{W}}_{0} (x)) + f(x) = 0,\,\,\,B\left( {{\mathbf{W}}_{0} \frac{{d{\mathbf{W}}_{0} }}{dx}} \right) = 0. $$

Auxiliary function $H(r)$ can be written as

23 $$ H(r) = rc_{1} + r^{2} c_{2} + r^{3} c_{3} + \ldots . $$

Here $$c_{1} ,$$$$c_{2} ,$$$$c_{3} , \ldots$$ are constants to be calculated, Eq. (20) can be written in the form

24 $$ {\mathbf{W}}(x,r,c_{i} ) = {\mathbf{W}}_{0} (x) + \sum\limits_{j \ge 1} {{\mathbf{W}}_{j} } (x,c_{i} )r^{j} ,\,\,\,\,i = 1,2, \ldots $$

Substituting Eq. (24) in Eq. (20) and comparing similar powers of $r$ we have the following system

25 $$ L({\mathbf{W}}_{1} (x)) = c_{1} N_{0} ({\mathbf{W}}_{0} (x)),\,\,\,B\left( {{\mathbf{W}}_{1} \frac{{d{\mathbf{W}}_{1} }}{dx}} \right) = 0, $$

26 $$ L({\mathbf{W}}_{j} (x) - {\mathbf{W}}_{j - 1} (x)) = c_{j} N_{0} ({\mathbf{W}}_{0} (x) + \sum\limits_{k = 1}^{j - 1} {c_{k} } [L({\mathbf{W}}_{j - k} (x)) + N_{j - k} ({\mathbf{W}}_{0} (x),{\mathbf{W}}_{1} (x), \ldots ,{\mathbf{W}}_{j - 1} (x))],\,\,\,B\left( {{\mathbf{W}}_{j} \frac{{d{\mathbf{W}}_{j} }}{dx}} \right) = 0,\,\,j = 1,2, \ldots $$

In Eq. (26) the term $N_{m} ({\mathbf{W}}_{0} (x),{\mathbf{W}}_{1} (x),...,{\mathbf{W}}_{m} (x))$ is the coefficient of $r^{m}$ in the expansion of

27 $$ N({\mathbf{W}}(x,r,c_{k} )) = N_{0} ({\mathbf{W}}_{0} (x)) + \sum\limits_{j \ge 1} {N_{j} ({\mathbf{W}}_{0} (x),{\mathbf{W}}_{1} (x), \ldots ,{\mathbf{W}}_{j} (x))} r^{j} ,\,\,k = 1,2, \ldots $$

Equation (22), Eqs. (25) and (26) can easily be computed for ${\mathbf{W}}_{j} (x),j \ge 0,$ the solution of Eq. (24) i.e., the convergence is exclusively depending on the constants $c_{1} ,c_{2} ,c_{3} , \ldots$ If at $r = 1$ it is convergent, then from Eq. (24) we have

28 $$ {\mathbf{W}}(x,c_{k} ) = {\mathbf{W}}_{0} (x) + \sum\limits_{j \ge 1} {{\mathbf{W}}_{j} (x,c_{k} )} . $$

In general, the solution of Eq. (19) is approximated by

29 $$ {\mathbf{W}}^{n} (x,c_{k} ) = {\mathbf{W}}_{0} (x) + \sum\limits_{j = 1}^{n} {{\mathbf{W}}_{j} (x,c_{k} )} ,\quad k = 1,2, \ldots ,n. $$

After substituting Eq. (29) in Eq. (19) we obtained the residual

30 $$ R(x,c_{k} ) = L({\mathbf{W}}^{n} (x,c_{k} )) + f(x) + N({\mathbf{W}}^{n} (x,c_{k} )),\quad k = 1,2, \ldots ,n. $$

When $R(x,c_{k} ) = 0$ i.e., the residual is zero, we obtained the exact solution ${\mathbf{W}}^{n} (x,c_{k} ),$ but, if $R(x,c_{k} ) \ne 0,$ that is, the residual is not zero then we can minimize as under

31 $$ J(c_{l} ) = \int\limits_{a}^{b} {R^{2} (x,c_{l} )dx} . $$

where $a$ and $b$ are constants, depending upon the under considering the problem, where $c_{1} ,c_{2} ,c_{3} ,...$ are unknown constants can be calculated from the conditions

32 $$ \frac{\partial J}{{\partial c_{l} }} = 0,\,\,l = 1,2, \ldots ,n. $$

Once we acquire the values of these constants, we get the approximate solution from Eq. (29). Besides the method proposed in this paper, there are other methods, sharing the same idea of optimization^73,74^.

### Basic notion of NIM

The New Iterative Method (NIM) is enlightened in this sub-section; let us consider the differential equation

33 $$ {\mathbf{W}}(x) = L({\mathbf{W}}(x)) + f(x) + N({\mathbf{W}}(x)). $$

where the linear operator is denoted by $L,$ $f(x)$ is known function, ${\mathbf{W}}(x)$ is the unknown function, $N({\mathbf{W}}(x))$ is the nonlinear operator. Suppose that the solution of the New Iterative Method of Eq. (33) is of the form

34 $$ {\mathbf{W}}(x) = \sum\limits_{i = 0}^{\infty } {{\mathbf{W}}_{i} } . $$

As $L$ is a linear operator, therefore

35 $$ L\left( {\sum\limits_{i = 0}^{\infty } {{\mathbf{W}}_{i} } } \right) = \sum\limits_{i = 0}^{\infty } {L({\mathbf{W}}_{i} } ). $$

The non-linear operator is given by^50^

36 $$ N\left( {\sum\limits_{i = 0}^{\infty } {{\mathbf{W}}_{i} } } \right) = N({\mathbf{W}}_{0} ) + \sum\limits_{i = 1}^{\infty } {\left\{ {N\left( {\sum\limits_{j = 0}^{i} {{\mathbf{W}}_{j} } } \right) - N\left( {\sum\limits_{j = 0}^{i - 1} {{\mathbf{W}}_{j} } } \right)} \right\}} = \sum\limits_{i = 0}^{\infty } {{\rm E}_{i} } . $$

where $$E_{0} = N({\mathbf{W}}_{0} )$$ and

37 $$ E_{i} = \left\{ {N\left( {\sum\limits_{j = 0}^{i} {{\mathbf{W}}_{j} } } \right) - N\left( {\sum\limits_{j = 0}^{i - 1} {{\mathbf{W}}_{j} } } \right)} \right\}. $$

Substituting Eqs. (34), (35) and (36) in Eq. (33) we obtained

38 $$ \sum\limits_{i = 0}^{\infty } {{\mathbf{W}}_{i} } = f(x) + \sum\limits_{j = 0}^{i} {L({\mathbf{W}}_{j} } ) + \sum\limits_{i = 0}^{\infty } {E_{i} } . $$

## Solution of the problem

OHAM’s solutions of velocity profile $({\mathbf{W}}_{{\mathbf{O}}} )$ and temperature distributions $(\Theta_{{\mathbf{\rm O}}} )$ up to second-order are as follows.

Zero order velocity problem

$$ w_{0}^{\prime\prime\prime\prime} (y) + A = 0,\, $$

$$ w_{0} ( - 1) = 0,w_{0} (1) = 1,w_{0}^{^{\prime\prime}} ( - 1) = 0,w_{0}^{^{\prime\prime}} (1) = 0 $$

Zero order velocity solution

$$ w_{0} = \frac{1}{24}(11.99 + \, 12 \, y + \, 0.012 \, y^{2} - \, 0.002\,y^{4} ). $$

First order velocity problem

$$ \begin{aligned} & - A - A\,c_{1} - B^{2} Mc_{1} w_{0}^{\prime } (y)\Theta_{0}^{^{\prime}} (y) + B^{2} c_{1} w_{0}^{\prime \prime } (y) - B^{2} Mc_{1} w_{0}^{\prime \prime } (y)\Theta_{0} (y) - (1 + c_{1} )w_{0}^{\prime \prime \prime \prime } (y) + w_{1}^{\prime \prime \prime \prime } (y) = 0,\, \\ & w_{1} ( - 1) = 0,w_{1} (1) = 0,w_{1}^{\prime \prime } ( - 1) = 0,w_{1}^{\prime \prime } (1) = 0 \\ \end{aligned} $$

First order velocity solution

$$ \begin{aligned} w_{1} & = (0.0162738 - 2.21762 \times 10^{ - 7} y - 0.0200319 \, y^{2} + 3.3564 \times 10^{ - 7} \, y^{3} + 0.00403785 \, y^{4} \\ & \quad - 1.25865 \times 10^{ - 7} y^{5} - 0.000279679 \, y^{6} + 1.19871 \times 10^{ - 8} y^{7} )/10080 \\ \end{aligned} $$

Second order velocity problem

$$ \begin{aligned}    &  - Ac_{2}  - B^{2} Mc_{2} w_{0}^{\prime } (y)\Theta _{0}^{\prime } (y) - B^{2} Mc_{1} w_{1}^{\prime } (y)\Theta _{0} (y) - B^{2} Mc_{1} w_{0}^{\prime } (y)\Theta _{1}^{\prime } (y) + B^{2} Mc_{2} w_{0}^{{\prime \prime }} (y) - B^{2} Mc_{2} w_{0}^{{\prime \prime }} (y)\Theta _{0}  \\     &  - B^{2} Mc_{1} w_{0}^{{\prime \prime }} (y)\Theta _{1}  + B^{2} Mc_{1} w_{1}^{{\prime \prime }} (y) - B^{2} Mc_{1} w_{1}^{{''}} (y)\Theta _{0}  - c_{2} w_{0}^{{\prime \prime \prime \prime }}  - (1 + c_{1} )w_{1}^{{\prime \prime \prime \prime }} (y) + w_{2}^{{\prime \prime \prime \prime }} (y) = 0, \\     & w_{2} ( - 1) = 0,w_{2} (1) = 0,w_{2}^{{\prime \prime }} ( - 1) = 0,w_{2}^{{\prime \prime }} (1) = 0 \\  \end{aligned}  $$

Second order velocity solution

$$ \begin{aligned} w_{2} & = (1/130767436800)\,\,( - 626.679 + 4.76938 \, y + 773.507 \, y^{2} \\ & \quad - \, 6.81482 \, y^{3} - 159.704 \, y^{4} + 2.04642 \, y^{5} + \, 13.5229 \, y^{6} \, \\ & \quad - \, 0.00104308 \, y^{7} - 0.647452 \, y^{8} \, + \, 0.0000600888 \, y^{9} \, \\ & \quad + \, 2.29052 \times 10^{ - 8} y^{10} + 1.29442 \times 10^{ - 8} y^{11} - 1.63414 \times 10^{ - 9} y^{12} \\ & \quad - 5.84644 \times 10^{ - 16} y^{13} - 5.92683 \times 10^{ - 13} y^{14} + 2.76606 \times 10^{ - 17} y^{15} ). \\ \end{aligned} $$

OHAM’s solutions of velocity profile ${\mathbf{(W}}_{{\mathbf{\rm O}}} {\mathbf{)}}$ up to second order are as under

39 $$ \begin{aligned}   {\mathbf{W}}_{O}  &  = w_{0}  + w_{1}  + w_{2}  \\    {\mathbf{W}}_{O}  &  = \frac{1}{{24}}\left( {11.99 + 12y + 0.012y^{2}  - 0.002y^{4} } \right)\, + \frac{\begin{gathered}   0.01627375 - 2.2176199 \times 10^{{ - 7}} y \hfill \\    - 0.02003192y^{2}  + 3.356 \times 10^{{ - 7}} y^{3}  \hfill \\    + 0.00403785y^{4}  - 1.258 \times 10^{{ - 7}} y^{5}  \hfill \\    - 0.00027967y^{6}  + 1.198 \times 10^{{ - 8}} y^{7}  \hfill \\  \end{gathered} }{{10080}}\frac{1}{{130767436800}}\\&\quad( - 626.67876360 + 4.76938223y + 773.50689375y^{2}  \\     & \quad  - 6.81481904y^{3}  - 159.7035486y^{4}  + 2.04641978y^{5}  + 13.52287073y^{6}  \\     & \quad  - 0.00104307y^{7}  - 0.64745228y^{8} \,\, + 0.00006008y^{9}  + 2.290 \times 10^{{ - 8}} y^{{10}}  \\     & \quad  + 1.294 \times 10^{{ - 8}} y^{{11}}  - 1.634 \times 10^{{ - 9}} y^{{12}} \, - 5.846 \times 10^{{ - 16}} y^{{13}}  - 5.93 \times 10^{{ - 13}} y^{{14}}  \\     & \quad  + 2.76605961 \times 10^{{ - 17}} y^{{15}} ). \\  \end{aligned}  $$

OHAM’s solutions of temperature distributions $(\Theta_{{\mathbf{\rm O}}} )$ up to second order are as under.

Zero order temperature problem:

$$ \begin{aligned} & \Theta_{0}^{\prime \prime } (y) = 0, \\ & \Theta_{0} ( - 1) = 0,\,\,\,\Theta_{0} (1) = 1, \\ \end{aligned} $$

Zero order temperature solution:

$$ \Theta_{0} = \frac{{\text{(1 + y)}}}{2} $$

First order temperature problem

$$ \begin{aligned} & - \lambda c_{1} (w_{0}^{\prime } (y))^{2} + \lambda c_{1} \Theta_{0} (w_{0}^{\prime } (y))^{2} - \frac{{\lambda c_{1} (w_{0}^{\prime \prime } (y))^{2} }}{{B^{2} }} - (1 + c_{1} )\Theta_{0}^{\prime \prime } (y) + \Theta_{1}^{\prime \prime } (y) = 0, \\ & \Theta_{1} ( - 1) = 0,\,\,\Theta_{1} (1) = 0, \\ \end{aligned} $$

First order temperature solution

$$ \begin{aligned} \Theta_{1} & = 0.000551146 \, (2.26526 \, + \, 0.00266089 \, y - 2.2655 \, y^{2} - \, 0.00296284 \, y^{3} \\ & \quad + 0.000300572 \, y^{4} + \, 0.000301946 \, y^{5} - \, 0.0000600061 \, y^{6} - \, 2.15692 \times 10^{ - 11} \, y^{7} \\ & \quad - \, 3.59459*10^{ - 8} \, y^{8} \, + \, 2.097 \times 10^{ - 12} \, y^{9} ). \\ \end{aligned} $$

Second order temperature problem

$$ \begin{aligned} & - \,\lambda c_{2} (w_{0}^{\prime } (y))^{2} + \lambda \,\Theta_{0} M\,c_{2} (w_{0}^{\prime } (y))^{2} + \lambda \,\Theta_{1} M\,c_{1} (w_{0}^{\prime } (y))^{2} - 2\lambda c_{1} w_{0}^{\prime } (y)w_{1}^{\prime } (y) + 2\lambda M\Theta_{0} c_{1} w_{0}^{\prime } (y)w_{1}^{\prime } (y) \\ & - \,\frac{{\lambda c_{2} (w_{0}^{\prime \prime } (y))^{2} }}{{B^{2} }} - \frac{{2\lambda c_{1} (w_{0}^{\prime \prime } (y))^{2} (w_{1}^{\prime \prime \prime } (y))^{2} }}{{B^{2} }} - c_{2} \Theta_{0}^{\prime \prime } (y) - (1 + c_{1} )\Theta_{1}^{\prime \prime } (y) + \Theta_{2}^{\prime \prime } (y) = 0, \\ & \Theta_{2} ( - 1) = 0,\,\,\Theta_{2} (1) = 0, \\ \end{aligned} $$

Second order temperature solution

40 $$ \begin{aligned} \Theta_{2} & = 1.24954 \times 10^{ - 12} \, (1.42663 \times 10^{6} - 3004.76 \, y \, - \, 1.42586 \times 10^{6} \, y^{2} \\ & \quad + 3422.68 \, y^{3} - \, 1005.75 \, y^{4} - \, 449.576 \, y^{5} \, + \, 260.975 \, y^{6} \, + 31.6642 \, y^{7} \\ & \quad - 23.5745 \, y^{8} + \, 0.00109017 \, y^{9} - \, 0.00985318 \, y^{10} + 1.12425 \times 10^{ - 6} y^{11} \\ & \quad + 3.42057 \times 10^{ - 10} \, y^{12} \, + \, 1.92831 \times 10^{ - 10} \, y^{13} - \, 2.41186 \times 10^{ - 11} \, y^{14} \\ & \quad - \, 1.1938 \times 10^{ - 17} \, y^{15} - \, 1.09947 \times 10^{ - 14} \, y^{16} \, + \, 5.65947 \times 10^{ - 19} \, y^{17} ). \\ \Theta_{O} & = \Theta_{0} + \Theta_{1} + \Theta_{2} . \\ \Theta_{O} & = \frac{1 + y}{2} + 0.00055114(2.26525631\, + 0.00266089y - 2.26549684y^{2} \\ & \quad - 0.00296283y^{3} + 0.00030057y^{4} \, + 0.00030194y^{5} - 0.00006000y^{6} \\ & \quad - 2.1569 \times 10^{ - 11} y^{7} - 3.59 \times 10^{ - 8} y^{8} \, + 2.097 \times 10^{ - 12} y^{9} ) + 1.24953 \times 10^{ - 12} \\ & \quad \,\,\,(1426633.172656 - 3004.76498y - 1425864.81y^{2} + 3422.675428y^{3} \\ & \quad - 1005.753209y^{4} - 449.57572y^{5} + 260.975092y^{6} + 31.6641830y^{7} \\ & \quad - 23.5745042y^{8} + 0.00109017y^{9} - 0.00985318y^{10} + 0.0000012y^{11} \\ & \quad + 3.421 \times 10^{ - 10} y^{12} + 1.9 \times 10^{ - 10} y^{13} - 2.412 \times 10^{ - 11} y^{14} - 1.19 \times 10^{ - 17} y^{15} \\ & \quad - 1.09 \times 10^{ - 14} y^{16} + 5.66 \times 10^{ - 19} y^{17} ). \\ \end{aligned} $$

NIM solutions of velocity profile ${\mathbf{(W}}_{{\mathbf{\rm N}}} {\mathbf{)}}$ and temperature distributions $(\Theta_{N} )$ up to second-order are as under.

Zero order NIM velocity problem

$$ w_{0}^{\prime\prime\prime\prime } (y) + A = 0,\, $$

$$ w_{0} ( - 1) = 0,w_{0} (1) = 1,w_{0}^{^{\prime\prime}} ( - 1) = 0,w_{0}^{^{\prime\prime}} (1) = 0 $$

Zero order velocity solution

$$ w_{0} = \frac{1}{24}(11.99 + \, 12 \, y + \, 0.012 \, y^{2} - \, 0.002 \, 0.1y^{4} ). $$

First order velocity problem

$$ B^{2} \left(\int {\iiint {(1 - M\Theta_{0} })\,D[w_{0} ,\,\{ y,\,2\} ]dydydydy} + \int {\iiint {D[(1 - M\Theta_{0} }),\,y]\,D[w_{0} ,y]dydydydy} \right), $$

First order velocity solution

$$ \begin{aligned} w_{1} & = {0}{\text{.01 (4}}{.16667} \times {10}^{ - 6} { (9}{\text{.99925 y}}^{4} \, - { 0}{\text{.00015 y}}^{5} \, - { 0}{\text{.666617 y}}^{6} \\ & \quad + {0}{\text{.0000214286 y}}^{7} {) + 1}{\text{.4881}} \times {10}^{ - 8} {\text{ y}}^{4} { (} - {105 + 0}{\text{.002 y (}} - {\text{21 + y}}^{2} {)))}{\text{.}} \\ \end{aligned} $$

Second order velocity problem

$$ \begin{aligned} & B^{2} \left(\int {\iiint {(1 - M(\Theta_{0} + \Theta_{1} )})\,D[w_{0} + w_{1} ,\,\{ y,\,2\} ]dydydydy} + \int {\iiint {D[(1 - M(\Theta_{0} + \Theta_{1} )}),\,y]\,D[w_{0} + w_{1} ,y]dydydydy} \right) \\ & - B^{2} \left(\int {\iiint {(1 - M\,\Theta_{0} })\,D[w_{0} ,\,\{ y,\,2\} ]dydydydy} + \int {\iiint {D[(1 - M\,\Theta_{0} }),\,y]\,D[w_{0} ,y]dydydydy}\right ), \\ \end{aligned} $$

Second order velocity solution

41 $$ \begin{aligned}   w_{2}  &  = 0.01{\text{ }}(4.30153 \times 10^{{ - 14}} {\text{ }}( - 3.63243 \times 10^{7} {\text{  }}y^{4} {\text{ }} + {\text{ }}21806.4{\text{  }}y^{5} {\text{ }} + {\text{ }}47.9844{\text{  }}y^{6}  \\     & \quad  + 687.205{\text{  }}y^{7}  - {\text{ }}2.58808{\text{  }}y^{8}  + {\text{ }}0.207826{\text{  }}y^{9}  - 1.41786 \times 10^{{ - 6}} {\text{  }}y^{{10}} {\text{ }} + {\text{ }}0.000156297{\text{ }}y^{{11}} {\text{ }} \\     & \quad  - 0.000019272{\text{ }}y^{{12}} {\text{ }} + {\text{ }}2.82084 \times 10^{{ - 8}} {\text{ }}y^{{13}}  - 1.24505 \times 10^{{ - 8}} {\text{ }}y^{{14}} {\text{ }} + {\text{  }}5.41557 \times 10^{{ - 13}} {\text{ }}y^{{15}} {\text{ }} \\     & \quad  - 2.11168 \times 10^{{ - 12}} {\text{ }}y^{{16}}  + {\text{ }}1.86727 \times 10^{{ - 16}} {\text{ }}y^{{17}}  - 4.12176 \times 10^{{ - 21}} {\text{ }}y^{{18}} ){\text{ }} + 1.40573 \times 10^{{ - 12}} {\text{ }} \\     & \quad (2.96384 \times 10^{7} {\text{ }}y^{4}  - 444.609{\text{  }}y^{5}  - 1.96638 \times 10^{6} {\text{ }}y^{6} {\text{ }} + {\text{ }}62.9985{\text{ }}y^{7}  - 352.891{\text{ }}y^{8} {\text{  }} \\     & \quad  + {\text{ }}0.0263995{\text{ }}y^{9}  - 0.0000183922{\text{ }}y^{{10}}  + {\text{ }}2.21701 \times 10^{{ - 6}} y^{{11}}  - 2.94627 \times 10^{{ - 7}} {\text{ }}y^{{12}} {\text{  }} \\     & \quad  + {\text{ }}8.63341 \times 10^{{ - 10}} {\text{ }}y^{{13}}  - 2.10313 \times 10^{{ - 10}} {\text{ }}y^{{14}}  + 9.09717 \times 10^{{ - 15}} {\text{ }}y^{{15}}  - 4.03859 \times 10^{{ - 14}} {\text{ }}y^{{16}} {\text{ }} \\     & \quad  + {\text{ }}3.65481 \times 10^{{ - 18}} {\text{ }}y^{{17}}  - 8.4084 \times 10^{{ - 23}} {\text{ }}y^{{18}} )) - 0.01{\text{ }}(4.16667 \times 10^{{ - 6}} (9.99925{\text{ }}y^{4} {\text{ }} \\     & \quad  - {\text{ }}0.00015{\text{ }}y^{5}  - 0.666617{\text{ }}y^{6} {\text{ }} + {\text{ }}0.0000214286{\text{ }}y^{7} ){\text{ }} + {\text{ }}1.4881 \times 10^{{ - 8}} {\text{ }}y^{4} {\text{ }}( - 105{\text{ }} + {\text{ }}0.002{\text{ }}y{\text{ }}( - 21{\text{ }} + {\text{ }}y^{2} ))). \\    W_{N}  &  = w_{0}  + w_{1}  + w_{2} .  \\  {\mathbf{W}}_{N}  &  = \frac{1}{{24}}\left( {11.99 + 12y + 0.012y^{2}  - 0.002y^{4} } \right) + 0.010(4.3015 \times 10^{{ - 14}} ( - 3.63242 \times 10^{7} y^{4}  \\     & \quad  + 21806.37819359y^{5}  + 47.98438444y^{6}  + 687.20471612y^{7}  - 2.5880756369y^{8}  \\     & \quad  + 0.20782637y^{9}  - 0.000001417858y^{{10}}  + 0.00015629y^{{11}}  - 0.00001927197y^{{12}}  \\     & \quad  + 2.820839 \times 10^{{ - 8}} y^{{13}}  - 1.245 \times 10^{{ - 8}} y^{{14}}  + 5.41556 \times 10^{{ - 13}} y^{{15}}  - 2.112 \times 10^{{ - 12}} y^{{16}}  \\     & \quad  + 1.86727 \times 10^{{ - 16}} y^{{17}}  - 4.12 \times 10^{{ - 21}} y^{{18}} )\, + 1.405728 \times 10^{{ - 12}} (2.9638395 \times 10^{7} y^{4}  \\     & \quad  - 444.609285y^{5}  - 1966384.449066y^{6} \, + 62.99847199y^{7}  - 352.89062504y^{8}  \\     & \quad  + 0.02639953y^{9}  - 0.00001839222y^{{10}}  + 0.00000222y^{{11}}  - 2.94626 \times 10^{{ - 7}} y^{{12}}  \\     & \quad  + 8.6334 \times 10^{{ - 10}} y^{{13}}  - 2.103 \times 10^{{ - 10}} y^{{14}}  + 9.0972 \times 10^{{ - 15}} y^{{15}}  - 4.038 \times 10^{{ - 14}} y^{{16}}  \\     & \quad  + 3.655 \times 10^{{ - 18}} y^{{17}}  - 8.41 \times 10^{{ - 23}} y^{{18}} )). \\  \end{aligned}  $$

Zero order temperature problem:

$$ \Theta_{0}^{^{\prime\prime}} (y) = 0, $$

$$ \Theta_{0} ( - 1) = 0,\,\,\,\Theta_{0} (1) = 1, $$

Zero order temperature solution:

$$ \Theta_{0} = \frac{{\text{(1 + y)}}}{2} $$

First order temperature problem

$$ - \lambda \left(\iint {(1 - M\Theta_{0} )(}D[w_{0} ,\,y])^{2} dydy + \frac{1}{{B^{2} }}\iint {(D[w_{0} ,\,\{ y,\,2\} ]})^{2} dydy\right) $$

First order temperature solution

$$ \begin{aligned} \Theta_{1} & = {0}{\text{.01 (}} - {3}{\text{.33333}} \times 10^{ - 6} \, y^{2} { (15 } - { 5 }y^{2} { + }y^{4} {) + 1/72 (} - \,{8}{\text{.99932 }}y^{2} - { 0}{\text{.0117741 }}y^{3} \, \\ & \quad - {5}{\text{.54955}} \times 10^{ - 6} \, y^{4} { + 0}{\text{.00119991 }}y^{5} { + 1}{\text{.53988}} \times 10^{ - 6} y^{6} - { 8}{\text{.57143}} \times 10^{ - 11} \, y^{7} \, \\ & \quad - {1}{\text{.42846}} \times 10^{ - 7} \, y^{8} { + 8}{\text{.33333}} \times 10^{ - 12} \, y^{9} {))}{\text{.}} \\ \end{aligned} $$

Second order temperature problem

$$ \begin{aligned} & - \lambda \left(\iint {(1 - M(\Theta_{0} + \Theta_{1} ))(}D[w_{0} + w_{1} ,\,y])^{2} dydy + \frac{1}{{B^{2} }}\iint {(D[w_{0} + w_{1} ,\,\{ y,\,2\} ]})^{2} dydy\right) \\ & - \left( - \lambda \left(\iint {(1 - M\,\Theta_{0} )(}D[w_{0} ,\,y])^{2} dydy + \frac{1}{{B^{2} }}\iint {(D[w_{0} ,\,\{ y,\,2\} ]})^{2} dydy\right)\right) \\ \end{aligned} $$

Second order temperature solution

$$ \begin{aligned} \Theta_{2} & = 0.01 \, (3.33333 \times 10^{ - 6} y^{2} \, (15 \, - 5 \, y^{2} \, + \, y^{4} ) \, + \, 1/72 \, (8.99932 \, y^{2} + \, 0.0117741 \, y^{3} \, \\ & \quad + 5.54955 \times 10^{ - 6} \, y^{4} - 0.00119991 \, y^{5} - 1.53988 \times 10^{ - 6} \, y^{6} + \, 8.57143 \times 10^{ - 11} \, y^{7} \, \\ & \quad { + }1.42846 \times 10^{ - 7} \, y^{8} - 8.33333 \times 10^{ - 12} \, y^{9} )) - 0.01 \, ( - 4.11032 \times 10^{ - 16} \, ( - 3.0409 \times 10^{14} \, y^{2} \\ & \quad - 3.9785 \times 10^{11} \, y^{3} - 1.97028 \times 10^{8} \, y^{4} \, + \, 4.03502 \times 10^{10} \, y^{5} \, + \, 5.17882 \times 10^{7} \, y^{6} \\ & \quad + 9.65402 \times 10^{6} \, y^{7} - 4.76695 \times 10^{6} \, y^{8} \, + \, 276.71 \, y^{9} - 2989.48 \, y^{10} \, + \, 0.340553 \, y^{11} \\ & \quad - 0.512215 \, y^{12} \, + \, 0.000146477 \, y^{13} - 7.29727 \times 10^{ - 6} \, y^{14} + \, 3.7686 \times 10^{ - 8} \, y^{15} \\ & \quad - 8.87289 \times 10^{ - 9} \, y^{16} \, + \, 6.62125 \times 10^{ - 12} \, y^{17} - 3.7137 \times 10^{ - 12} \, y^{18} \, + \, 3.50981 \times 10^{ - 16} \, y^{19} \, \\ & \quad - 5.29186 \times 10^{ - 16} \, y^{20} \, + \, 7.58125 \times 10^{ - 20} \, y^{21} - 3.62766 \times 10^{ - 24} \, y^{22} \, + \, 5.79681 \times 10^{ - 29} \, y^{23} ) \, \\ & \quad { + }1.25261 \times 10^{ - 6} \, y^{2} \, (9.3555 \times 10^{ - 8} \, y^{4} - 4.95 \times 10^{ - 7} \, y^{2} \, (5040 - - 2005.92 \, y^{2} - 0.00036 \, y^{3} \, \\ & \quad - 0.899933 \, y^{4} \, + \, 0.000042 \, y^{5} ) \, + \, 4. \times 10^{ - 6} \, (9979200 - 3.30977 \times 10^{6} \, y^{2} \, 0.49896 \, y^{3} \, \\ & \quad { + }657536. \, y^{4} + 0.283932 \, y^{5} + 590.986 \, y^{6} - 0.0274659 \, y^{7} + 0.153977 \, y^{8} - 0.0000151189 \, y^{9} + \, 3.78 \times 10^{ - 10} \, y^{10} ))). \\ \end{aligned} $$

NIM’s temperature solution up to second order as under

42 $$ \begin{aligned} \Theta_{N} & = \Theta_{0} + \Theta_{1} + \Theta_{2} . \\ \Theta_{N} & = \frac{1 + y}{2} + 0.01(0.0000033y^{2} \left( {15 - 5y^{2} + y^{4} } \right) + \frac{1}{72}(8.999324999999y^{2} + 0.0117741y^{3} \\ & \quad + 0.00000554955y^{4} - 0.00119991027y^{5} \, - 0.00000153y^{6} + 8.57142857 \times 10^{ - 11} y^{7} \\ & \quad + 1.42846428 \times 10^{ - 7} y^{8} - 8.333 \times 10^{ - 12} y^{9} )) + 0.01( - 0.00000333333y^{2} \left( {15 - 5y^{2} + y^{4} } \right) \\ & \quad + \frac{1}{72}( - 8.99932499y^{2} - 0.0117741y^{3} - 0.00000554955y^{4} + 0.00119991027y^{5} \\ & \quad + 0.00000153y^{6} - 8.57142857 \times 10^{ - 11} y^{7} - 1.42846428 \times 10^{ - 7} y^{8} + 8.33 \times 10^{ - 12} y^{9} )) \\ & \quad - 0.01( - 4.110317 \times 10^{ - 16} ( - 3.040 \times 10^{14} y^{2} \, - 3.97850437 \times 10^{11} y^{3} - 1.970276 \times 10^{8} y^{4} \\ & \quad + 4.035019 \times 10^{10} y^{5} + 5.17881640 \times 10^{7} y^{6} + 9654017.1191y^{7} - 4766951.2632218y^{8} \\ & \quad + 276.71033281y^{9} - 2989.4806939058y^{10} + 0.3405532095y^{11} - 0.512214714563y^{12} \\ & \quad + 0.0001464765y^{13} - 0.000007297266y^{14} + 3.76860 \times 10^{ - 8} y^{15} - 8.8728907 \times 10^{ - 9} y^{16} \\ & \quad + 6.62125 \times 10^{ - 12} y^{17} - 3.713703 \times 10^{ - 12} y^{18} + 3.50981 \times 10^{ - 16} y^{19} - 5.291859 \times 10^{ - 16} y^{20} \\ & \quad + 7.58125 \times 10^{ - 20} y^{21} - 3.627664 \times 10^{ - 24} y^{22} + 5.79681 \times 10^{ - 29} y^{23} ) + 0.00000125260y^{2} \\ & \quad (9.35550 \times 10^{ - 8} y^{4} - 4.9500000 \times 10^{ - 7} y^{2} (5040 - 2005.920756y^{2} - 0.00036y^{3} \\ & \quad - 0.89993250y^{4} + 0.000042y^{5} ) + 0.000004(9979200 - 3309769.2474y^{2} \\ & \quad - 0.49896y^{3} + 657535.6116252935y^{4} + 0.28393208y^{5} + 590.9858955055y^{6} \\ & \quad - 0.02746591y^{7} + 0.153976895322y^{8} \, - 0.00001512y^{9} + 3.780 \times 10^{ - 10} y^{10} ))). \\ \end{aligned} $$

### Volume flux

The volume flux $Q$ in the non-dimensional form as is under

43 $$ Q = \mathop \int \limits_{ - 1}^{1} {\mathbf{W}}{\text{d}}y. $$

Substituting Eqs. (39) and (41) in (43) we obtained

44 $$ \begin{aligned}   Q_{O}  &  = 1. - \frac{{4A}}{{15}} + 0.107937AB^{2}  - 0.043645AB^{4}  - 0.06666718B^{2} M - 0.05396867AB^{2} M \\     & \quad  + 0.0269263B^{4} M + 0.043645244AB^{4} M\, - 0.01346315B^{4} M^{2}  - 0.0114808AB^{4} M^{2}  \\     & \quad  - 0.00396381A^{3} M\lambda  - 0.010242AB^{2} M\lambda \, - 0.00103101A^{3} B^{2} M\lambda  + 0.00118B^{2} M^{2} \lambda  \\     & \quad  + 0.0051212AB^{2} M^{2} \lambda  + 0.002A^{2} B^{2} M^{2} \lambda  + 0.00051551A^{3} B^{2} M^{2} \lambda . \\  \end{aligned}  $$

45 $$ \begin{gathered} Q_{N} = 1 - \frac{4A}{{15}} + \frac{{AB^{2} }}{126} + \frac{{AB^{4} }}{5184} - \frac{{B^{2} M}}{240} - \frac{1}{252}AB^{2} M - \frac{{B^{4} M}}{10080} - \frac{{AB^{4} M}}{5184} + \frac{{B^{4} M^{2} }}{20160} + \frac{{1093AB^{4} M^{2} }}{19958400} \hfill \\ \,\,\,\,\,\,\,\,\,\,\, + \frac{{16073A^{3} M\lambda }}{129729600} + \frac{{AB^{2} M\lambda }}{4320} + \frac{{1232423A^{3} B^{2} M\lambda }}{108972864000} + \frac{{529AB^{4} M\lambda }}{79833600} + \frac{{5474983A^{3} B^{4} M\lambda }}{14820309504000} - \frac{{B^{2} M^{2} \lambda }}{40320} \hfill \\ \,\,\,\,\,\,\,\,\,\,\, - \frac{{AB^{2} M^{2} \lambda }}{8640} - \frac{{8243A^{2} B^{2} M^{2} \lambda }}{778377600} - \frac{{1232423A^{3} B^{2} M^{2} \lambda }}{217945728000}\, - \frac{{B^{4} M^{2} \lambda }}{290304} - \frac{{529AB^{4} M^{2} \lambda }}{79833600} - \frac{{35551A^{2} B^{4} M^{2} \lambda }}{36324288000} \hfill \\ \,\,\,\,\,\,\,\,\,\,\,\, - \frac{{5474983A^{3} B^{4} M^{2} \lambda }}{14820309504000} + \frac{{B^{4} M^{3} \lambda }}{580608} + \frac{{4079AB^{4} M^{3} \lambda }}{2075673600}\, + \frac{{35551A^{2} B^{4} M^{3} \lambda }}{72648576000} + \frac{{792750407A^{3} B^{4} M^{3} \lambda }}{7602818775552000}. \hfill \\ \end{gathered} $$

### Average velocity

The average velocity of the couple stress is represented and defined as

46 $$ \overline{{\mathbf{W}}} = \frac{Q}{d}. $$

The non-dimensional form of Eq. (46) coincides with the flow rate given in Eqs. (44) and (45).

### Shear stress

The dimensionless shear stress is symbolized and defined as follows

47 $$ t_{{\ominus }} = - \mu \,D\left[ {{\mathbf{W}},y} \right],y = 1. $$

In this paper, only tables and graphs are given for shear stress of both methods, because these equations are very lengthy in this case, the minus sign^75^ is due to the upper plate facing the negative $y - {\text{direction}}$ of the coordinate system. Here ${\text{Q}}_{{\text{O}}} \,\,{\text{and}}\,\,{\text{Q}}_{{\text{N}}} ,$ are the volume fluxes obtained using OHAM and NIM respectively.

## Results and discussion

**Figure 2:**
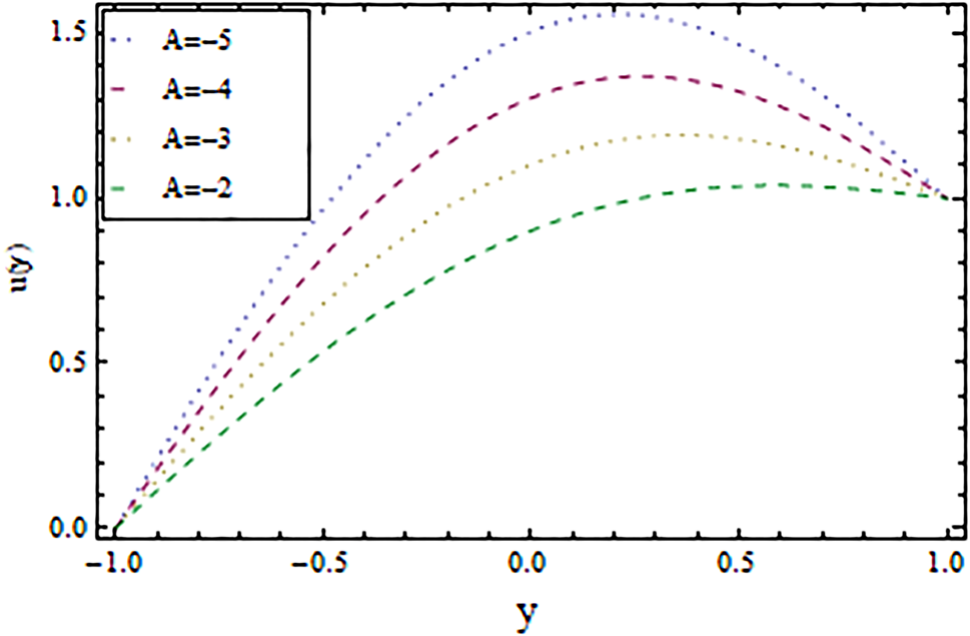
Velocity profile for $M = 0.0002,B = 0.3\,{\text{and}}\,\lambda = 3$ using OHAM.

**Figure 3:**
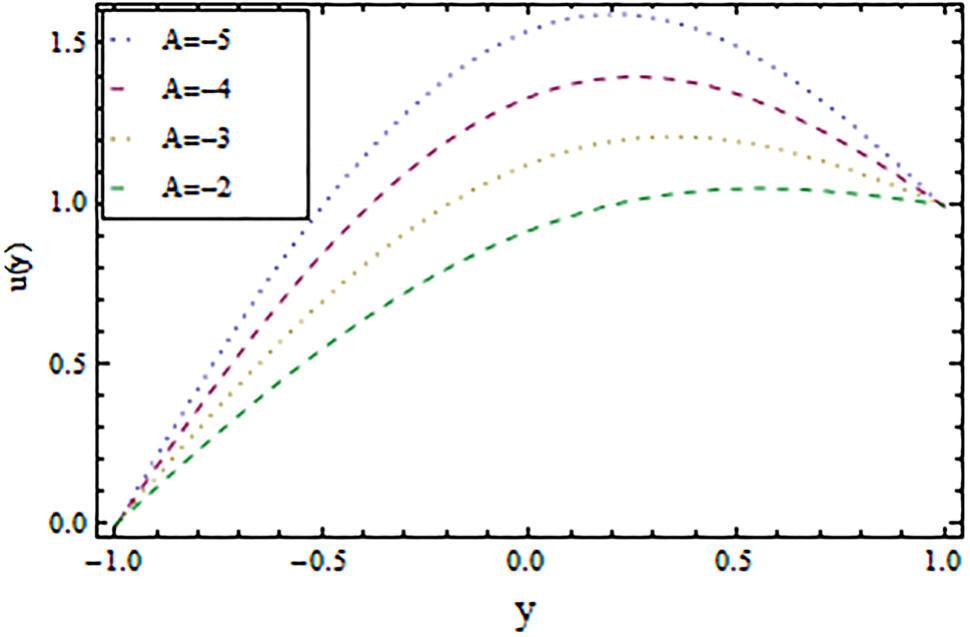
Velocity profile for $M = 0.0002,B = 0.3\,{\text{and}}\,\lambda = 3$ using NIM.

**Figure 4:**
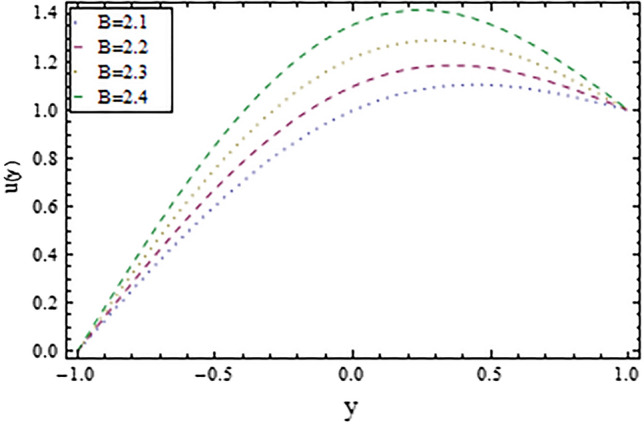
Velocity profile for $A = - 1,M = 0.0015\,{\text{and}}\,\lambda = 2$ using OHAM.

**Figure 5:**
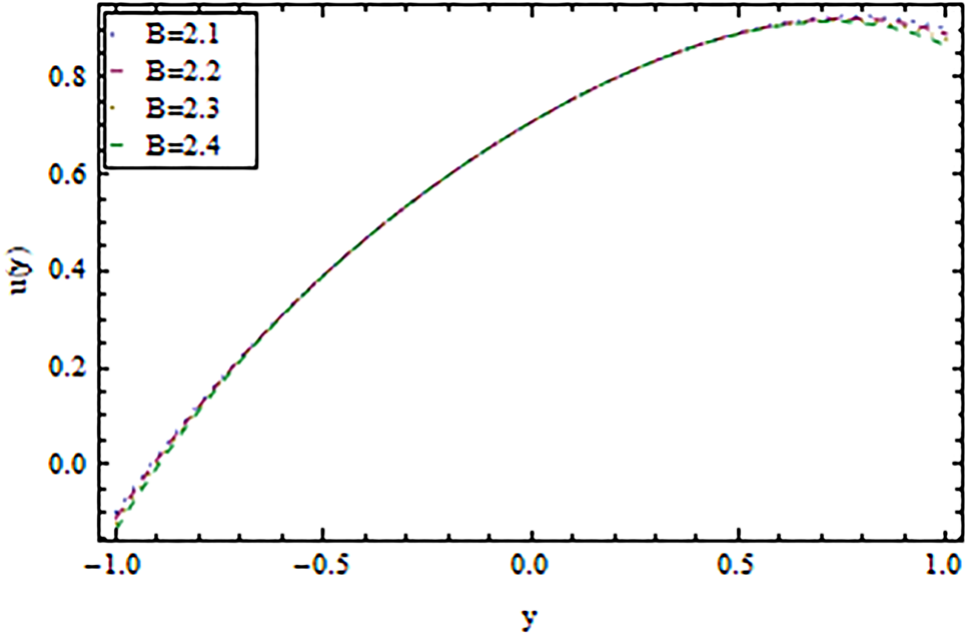
Velocity profile for $A = - 1,M = 0.0015\,{\text{and}}\,\lambda = 2$ using NIM.

**Figure 6:**
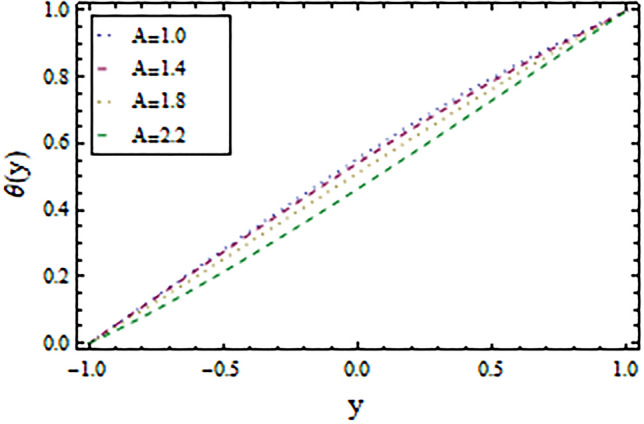
Temperature distribution for $M = 1,B = 3\,and\,\lambda = 1$ using OHAM.

**Figure 7:**
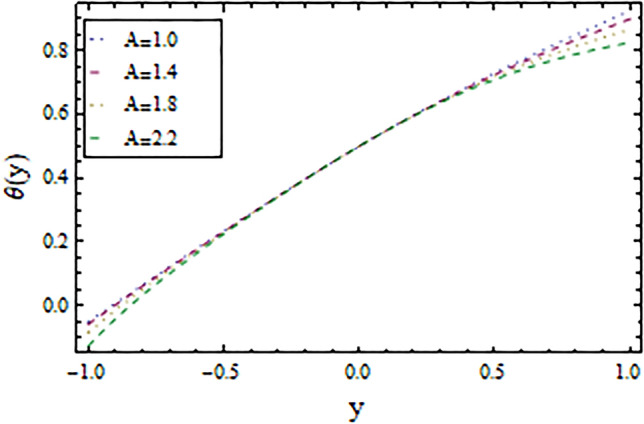
Temperature distribution for $M = 1,B = 3\,and\,\lambda = 1$ using NIM.

**Figure 8:**
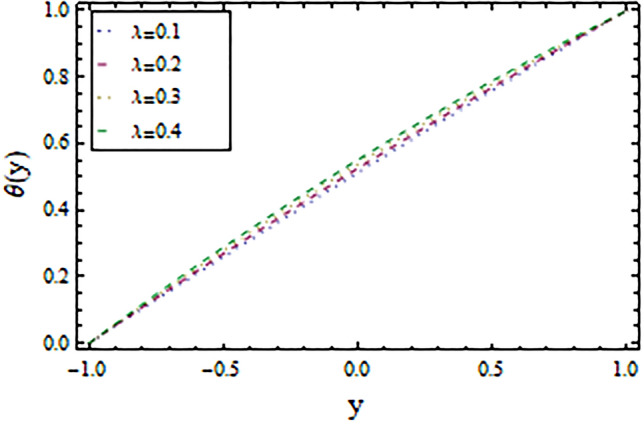
Temperature distribution for $A = 0.002,B = 0.01\,{\text{and}}\,M = 0.002$ using OHAM.

**Figure 9:**
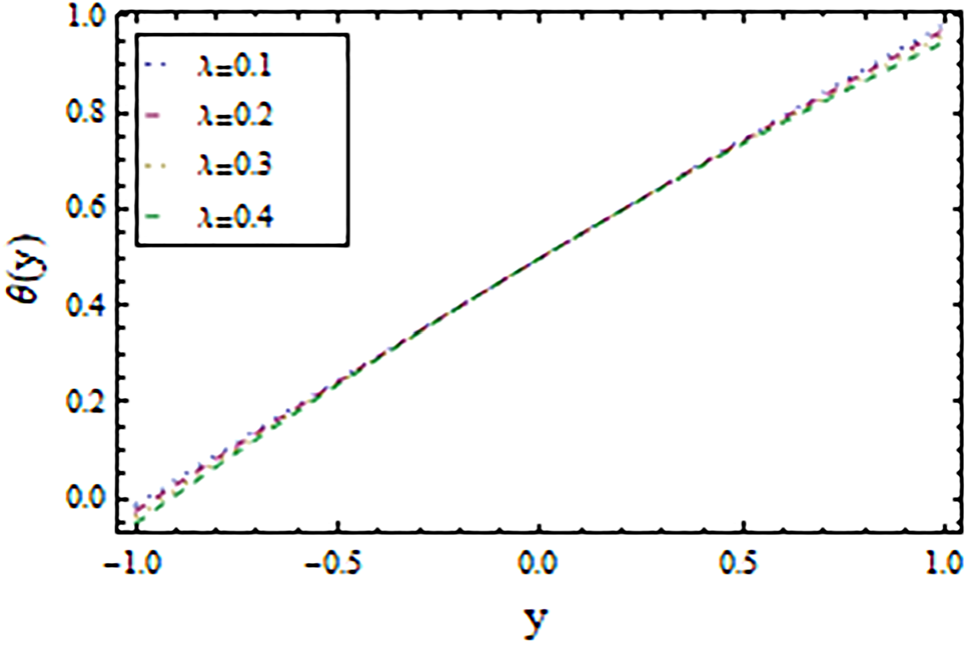
Temperature distribution for $A = 0.002,B = 0.01\,{\text{and}}\,M = 0.002$ using NIM.

**Figure 10:**
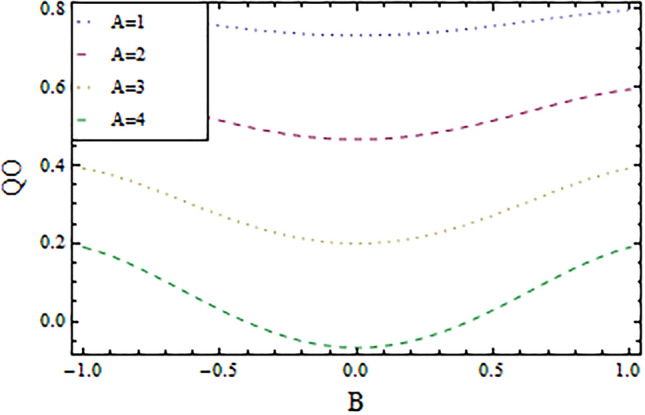
Volume flux of generalized plane Couette flow for $M = 0.0005,\lambda = 1$ using OHAM.

**Figure 11:**
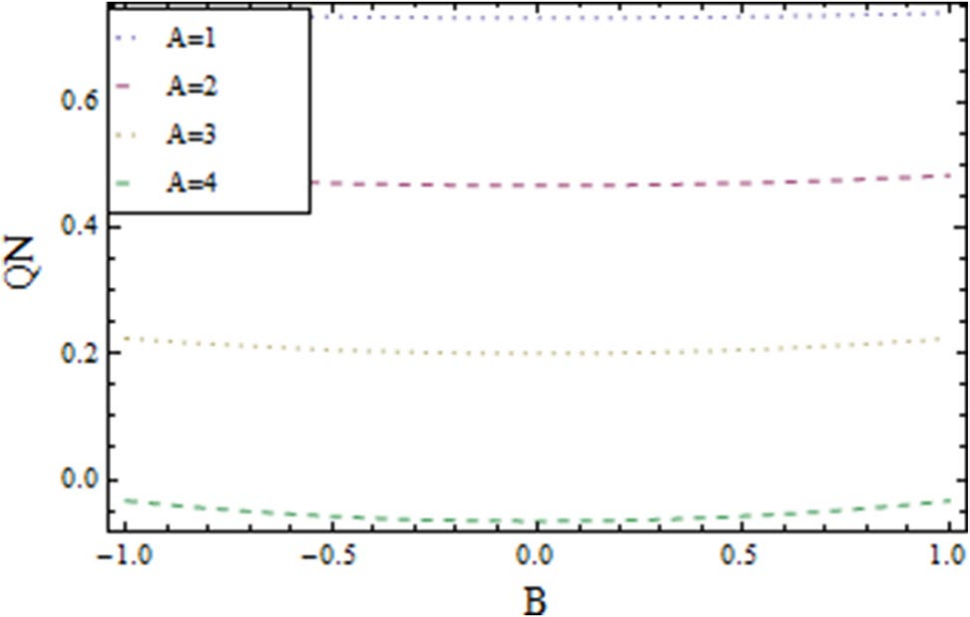
Volume flux of generalized plane Couette flow for $M = 0.0005,\lambda = 1$ using NIM.

**Figure 12:**
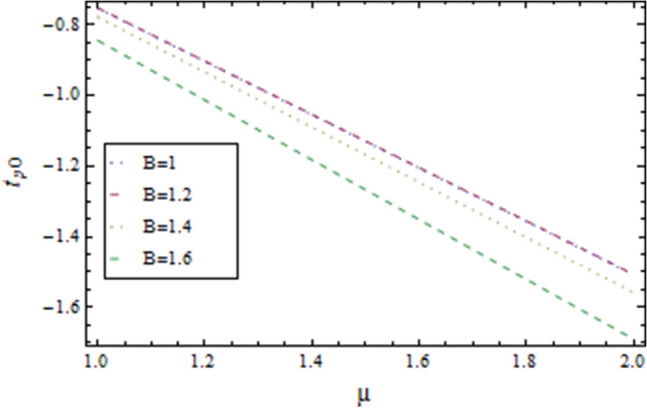
Shear Stress for $M = 0.0005,\lambda = 0.1\,{\text{and }}A = 1$ using OHAM.

**Figure 13:**
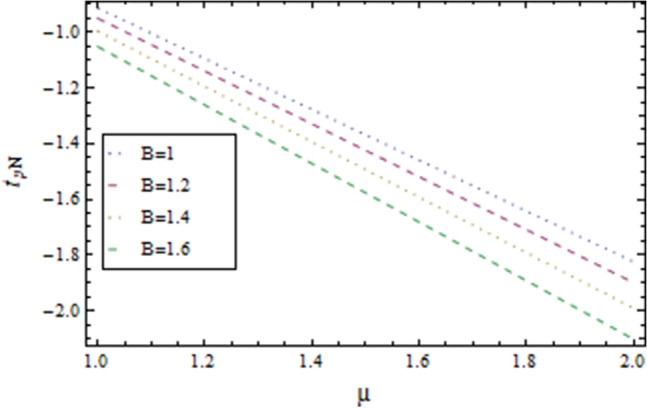
Shear Stress for $M = 0.0005,\lambda = 0.1\,{\text{and }}A = 1$ using NIM.

In the present work, we have investigated the variation of the velocity profile and temperature distribution on different parameters such as $A,\,B,M\,{\text{and}}\,\lambda .$ Tables 1 and 2 shows solutions of OHAM and NIM for velocity profile, temperature distributions and their residual of both methods respectively. Tables 3, 4, 5 and 6 illustrated the differences in velocity profile and temperature distributions using different parameters for both methods. Figures 2, 3, 4 and 5 demonstrates the velocity profile and temperature distributions of both methods for different parameters, which are closed to each other, the velocity of the fluid increases as the fluid move from stationary plate towards moving plate also the temperature of the fluid increases when moves from stationary plate to the moving plate. In Figs. 6 and 7 the effect of the parameter $A$ on the temperature is shown of both methods, there is an inverse relation between parameter $A$ and temeperature distribution. Figures 8 and 9 are plotted to see the effect of the parameter $\lambda$ on the temperature distribution, attained by two reliable techniques, OHAM and NIM, respectively. $\lambda$ is actually a non-dimensional quantity termed as the Brinkman number and is usually denoted by $B_{r} .$ As can be seen in Fig. 8, the increase in $\lambda$ increases the temperature of the fluid due to viscous heating of the fluid. Figure 9 also depicts the direct relationship between the temperature distribution and the dimensionless parameter $\lambda$.This implies that more and more heat is generated from the viscous heating of the fluid over the heat transfer from the heated wall to the fluid as shown in these two graph. The comparison of volume flux of both methods using different parameters are shown in Figs. 10 and 11, which are in good agreement, also in Figs. 12 and 13 we have observed the behaviour of shear stress $\tau_{p}$ in the generalized plane Couette flow by changing the values of the parameter $B.$ The shear stress $\tau_{p}$ and $B$ are inversely related as clear from these figures.

**Table 1:** For $$A = 0.002, \, B = 0.1, \, M = 0.00015{\text{ and }}\lambda = 0.01.$$

y	OHAM ${\mathbf{W}}_{{\mathbf{\rm O}}}$	Residual OHAM ${\mathbf{W}}_{{\mathbf{\rm O}}}$	NIM ${\mathbf{W}}_{N}$	Residual NIM ${\mathbf{W}}_{N}$
− 1	8.89043 × 10^–18^	1.12842 × 10^–12^	3.73359 × 10^–7^	− 3.4207 × 10^–11^
− 0.9	0.0499339	3.1777 × 10^–12^	0.0499339	− 2.25611 × 10^–11^
− 0.8	0.0998697	4.14403 × 10^–12^	0.0998694	− 1.41 × 10^–11^
− 0.7	0.149809	4.2532 × 10^–12^	0.149808	− 8.23091 × 10^–12^
− 0.6	0.199753	3.74221 × 10^–12^	0.199753	− 4.39301 × 10^–12^
− 0.5	0.249704	2.84421 × 10^–12^	0.249703	− 2.07242 × 10^–12^
− 0.4	0.299663	1.7756 × 10^–12^	0.299661	− 8.15201 × 10^–13^
− 0.3	0.349629	7.24956 × 10^–13^	0.349628	− 2.38387 × 10^–13^
− 0.2	0.399605	− 1.55901 × 10^–13^	0.399603	− 3.89675 × 10^–14^
− 0.1	0.44959	− 7.5891 × 10^–13^	0.449588	− 8.79559 × 10^–16^
0	0.499585	− 1.02476 × 10^–12^	0.499583	0
0.1	0.54959	− 9.45877 × 10^–13^	0.549588	− 7.13598 × 10^–15^
0.2	0.599605	− 5.67415 × 10^–13^	0.599603	− 8.90145 × 10^–14^
0.3	0.649629	1.3748 × 10^–14^	0.649628	− 4.07271 × 10^–13^
0.4	0.699663	6.52013 × 10^–13^	0.699661	− 1.21544 × 10^–12^
0.5	0.749704	1.15806 × 10^–12^	0.749703	− 2.85394 × 10^–12^
0.6	0.799753	1.30586 × 10^–12^	0.799753	− 5.74305 × 10^–12^
0.7	0.849809	8.41645 × 10^–13^	0.849808	− 1.0374 × 10^–11^
0.8	0.89987	− 5.05091 × 10^–13^	0.899869	− 1.72977 × 10^–11^
0.9	0.949934	− 3.0086 × 10^–12^	0.949934	− 2.7112 × 10^–11^
1	1	− 6.93191 × 10^–12^	1	− 4.04468 × 10^–11^

**Table 2:** For $$A = 0.002, \, B = 0.1, \, M = 0.00015{\text{ and }}\lambda = 0.01.$$

y	OHAM $\Theta_{O}$	Residual $\Theta_{O}$	NIM $\Theta_{N}$	Residual $\Theta_{N}$
− 1	3.23023 × 10^–19^	− 6.12875 × 10^–11^	− 0.0012488	− 6.80704 × 10^–12^
− 0.9	0.0502373	− 6.04614 × 10^–11^	0.0489884	− 3.15643 × 10^–12^
− 0.8	0.10045	− 5.75964 × 10^–11^	0.0992006	− 1.19945 × 10^–12^
− 0.7	0.150637	− 5.29869 × 10^–11^	0.149388	− 2.83398 × 10^–13^
− 0.6	0.2008	− 4.70084 × 10^–11^	0.19955	5.29563 × 10^–14^
− 0.5	0.250937	− 4.00936 × 10^–11^	0.249688	1.12833 × 10^–13^
− 0.4	0.30105	− 3.27016 × 10^–11^	0.2998	7.56639 × 10^–14^
− 0.3	0.351137	− 2.52864 × 10^–11^	0.349888	3.0352 × 10^–14^
− 0.2	0.4012	− 1.82654 × 10^–11^	0.39995	6.17562 × 10^–15^
− 0.1	0.451238	− 1.19926 × 10^–11^	0.449987	1.72171 × 10^–16^
0	0.50125	− 6.73623 × 10^–12^	0.5	0
0.1	0.551238	− 2.66376 × 10^–12^	0.549987	1.57036 × 10^–15^
0.2	0.601201	1.65194 × 10^–13^	0.59995	2.09047 × 10^–14^
0.3	0.651138	1.79791 × 10^–12^	0.649887	9.98993 × 10^–14^
0.4	0.701051	2.37925 × 10^–12^	0.6998	3.05928 × 10^–13^
0.5	0.750938	2.1346 × 10^–12^	0.749687	7.25391 × 10^–13^
0.6	0.800801	1.34631 × 10^–12^	0.79955	1.45156 × 10^–12^
0.7	0.850638	3.2485 × 10^–13^	0.849387	2.56731 × 10^–12^
0.8	0.90045	− 6.22983 × 10^–13^	0.899199	4.12343 × 10^–12^
0.9	0.950238	− 1.22644 × 10^–12^	0.948986	6.11371 × 10^–12^
1	1	− 1.28067 × 10^–12^	0.998748	8.44775 × 10^–12^

**Table 3:** For $$\lambda = \, 0.4, \, M \, = \, 0.0002, \, B \, = \, 0.3{\text{ and }}A \, = { 0}.4.$$

y	OHAM ${\mathbf{W}}_{{\mathbf{\rm O}}}$	NIM ${\mathbf{W}}_{N}$	Difference	OHAM $\Theta_{O}$	NIM $\Theta_{N}$	Difference
− 1	1.81941 × 10^–17^	0.000701923	0.000701923	3.52094 × 10^–18^	− 0.104916	0.104916
− 0.9596	0.0150044	0.0154161	0.0004117	0.0270457	− 0.0780229	0.1050686
− 0.7576	0.0908655	0.0900099	0.0008556	0.160743	0.0548985	0.1058445
− 0.5556	0.170038	0.168218	0.00182	0.290752	0.183969	0.106783
− 0.3535	0.25465	0.252164	0.002486	0.414929	0.306888	0.108041
− 0.1515	0.346058	0.343204	0.002854	0.530855	0.421123	0.109732
0.1515	0.497558	0.494704	0.002854	0.685768	0.57253	0.113238
0.3535	0.60815	0.605664	0.002486	0.775479	0.659224	0.116255
0.5556	0.725638	0.723818	0.00182	0.854926	0.735131	0.119795
0.7576	0.848466	0.84761	0.000856	0.925329	0.801542	0.123787
0.9596	0.974604	0.975016	0.000412	0.988222	0.860095	0.128127
1	1	1.0007	0.0007	1	0.870976	0.129024

**Table 4:** For $$\lambda \, = \, 0.1, \, M \, = \, 0.0015,B \, = \, 0.4{\text{ and }}A \, = { 0}.3.$$

y	OHAM ${\mathbf{W}}_{{\mathbf{\rm O}}}$	NIM ${\mathbf{W}}_{N}$	Difference	OHAM $\Theta_{O}$	NIM $\Theta_{N}$	Difference
− 1	− 9.49867 × 10^–18^	0.000935499	0.00093549	1.00273 × 10^–18^	− 0.0156358	0.0156358
− 0.8586	0.0575326	0.0572135	0.0003191	0.0745209	0.0587124	0.015809
− 0.6566	0.14116	0.139391	0.001769	0.180352	0.164301	0.016051
− 0.4545	0.228075	0.225251	0.002824	0.285345	0.269028	0.016317
− 0.2525	0.319477	0.315985	0.003492	0.389179	0.372553	0.016626
− 0.0505	0.416218	0.412441	0.003777	0.491696	0.474701	0.016995
0.0505	0.466718	0.462941	0.003777	0.542407	0.5252	0.017207
0.2525	0.571977	0.568485	0.003492	0.642664	0.624973	0.017691
0.4545	0.682575	0.679751	0.002824	0.741324	0.723069	0.018255
0.6566	0.79776	0.795991	0.001769	0.838446	0.819544	0.018902
0.8586	0.916133	0.915813	0.00032	0.933991	0.914369	0.019622
1	1	1.00094	0.00094	1	0.979836	0.020164

**Table 5:** For $$\lambda \, = \, 0.5, \, M \, = \, 0.0009,B \, = \, 0.3{\text{ and }}A \, = { 0}.3.$$

y	OHAM ${\mathbf{W}}_{{\mathbf{\rm O}}}$	NIM ${\mathbf{W}}_{N}$	Difference	OHAM $\Theta_{O}$	NIM $\Theta_{N}$	Difference
− 1	− 9.52025 × 10^–18^	0.000525569	0.00052556	− 1.52918 × 10^–18^	− 0.0982724	0.0982724
− 0.9192	0.0326247	0.032725	0.0001003	0.0538495	− 0.0448806	0.0987301
− 0.7172	0.115117	0.114315	0.000802	0.185915	0.0860338	0.0998812
− 0.5152	0.200446	0.198966	0.00148	0.313313	0.212163	0.10115
− 0.3131	0.290121	0.288188	0.001933	0.434406	0.331757	0.102649
− 0.1111	0.38504	0.382874	0.002166	0.547382	0.442927	0.104455
0.1111	0.49614	0.493974	0.002166	0.660854	0.553993	0.106861
0.3131	0.603221	0.601288	0.001933	0.753552	0.644098	0.109454
0.5152	0.715646	0.714166	0.00148	0.836464	0.724035	0.112429
0.7172	0.832317	0.831515	0.000802	0.910224	0.794475	0.115749
0.9192	0.951825	0.951925	1 × 10^–4^	0.975846	0.856495	0.119351
1	1	1.00053	0.00053	1	0.879149	0.120851

**Table 6:** For $$\lambda \, = \, 0.2, \, M \, = \, 0.001,B \, = \, 0.9{\text{ and }}A \, = { 0}{\text{.2}}{.}$$.

y	OHAM ${\mathbf{W}}_{{\mathbf{\rm O}}}$	NIM ${\mathbf{W}}_{N}$	Difference	OHAM $\Theta_{O}$	NIM $\Theta_{N}$	Difference
− 1	8.2188 × 10^–18^	0.00322804	0.00322804	1.5425 × 10^–18^	− 0.0230161	0.0230161
− 0.8182	0.0815681	0.0804385	0.0011296	0.0987602	0.0751311	0.023629
− 0.6162	0.173241	0.168495	0.004746	0.206869	0.182716	0.024153
− 0.4141	0.266937	0.259711	0.007226	0.313261	0.28869	0.024571
− 0.0101	0.363209	0.354523	0.008686	0.417743	0.392802	0.024941
− 0.2121	0.462484	0.453288	0.009196	0.520266	0.494947	0.025319
0.0101	0.472584	0.463388	0.009196	0.530407	0.505047	0.02536
0.2121	0.575309	0.566623	0.008686	0.630651	0.604839	0.025812
0.4141	0.681037	0.67381	0.007227	0.728712	0.702322	0.02639
0.6162	0.78944	0.784695	0.004745	0.824541	0.797395	0.027146
0.8182	0.899768	0.898638	0.00113	0.917969	0.889835	0.028134
1	1	1.00323	0.00323	1	0.970739	0.029261

## Conclusion

In this paper, the generalized plane Couette flow of couple stress fluids between two parallel plates has been investigated using OHAM and NIM. Emplying the said methods the strongly nonlinear and coupled system of differential equations are explored for velocity, temperature distributions, average velocity, volumetric flow rate and shear stress on the plates. The results obtained by these methods are in the form of infinite power series. The results of OHAM and NIM are compared numerically as well as graphically and a tremendous agreement is attained. The usefulness of these methods is clear from this research work.

## List of symbols

$$W$$: Velocity vector
$$\Theta$$: Dimensional temperature
$$c_{p}$$: Specific heat
$$\rho$$: Constant density
$\Theta_{0}$: Lower plate temperature
$$L$$: Gradient of $$W$$
$$v$$: Dimensional velocity
$$p$$: Dynamic pressure
$$\mu_{0}$$: Reference viscosity
$$\frac{D}{Dt}$$: Material derivative
$\mu$: Dimensional coefficient of viscosity
$$f$$: Body force
$$V$$: Reference velocity
$$\Theta^{*}$$: Dimensionless temperature
$\tau$: Cauchy stress tensor
$$\kappa$$: Thermal conductivity
$\Theta_{1}$: Upper plate temperature
$\eta$: Couple stress parameter
$$v^{*}$$: Dimensionless velocity
$$I$$: Unit tensor
$A_{1}$: First Rivilin–Ericksen tensor
$\tau$: Cauchy stress tensor
$\mu^{*}$: Dimensionless coefficient of viscosity
$$B$$: Dimensionless parameter
